# Chronotype, chrononutrition and glucose tolerance among prediabetic individuals: research protocol for a prospective longitudinal study Chrono-DM™

**DOI:** 10.1186/s12875-022-01815-7

**Published:** 2022-08-04

**Authors:** Guey Yong Chong, Satvinder Kaur, Ruzita Abd Talib, See Ling Loy, Hui Yin Tan, Sarjit Singh Harjit Singh, Rosmiza Binti Abdullah, Hanisah Binti Mahmud, Woan Yie Siah, Hui Chin Koo

**Affiliations:** 1 Faculty of Applied Sciences, Tunku Abdul Rahman University College, Kuala Lumpur, Malaysia; 2 Faculty of Applied Sciences, UCSI University, Kuala Lumpur, Malaysia; 3 Nutritional Sciences Program, Centre for Community Health Studies (ReaCH), Faculty of Health Sciences, The National University of Malaysia, Kuala Lumpur, Malaysia; 4 Department of Reproductive Medicine, KK Women’s and Children’s Hospital, Singapore, Singapore; 5 Duke-NUS Medical School, Singapore, Singapore; 6 Hospital Serdang, Sepang, Selangor Malaysia; 7 Klinik Kesihatan Batu Berendam, Pejabat Kesihatan Daerah Melaka Tengah, Melaka, Malaysia

**Keywords:** Chrononutrition, Chronotype, Glycemic outcomes, Glucose tolerance, Meal timing, Prediabetes, Longitudinal study

## Abstract

**Background:**

Chronotype and chrononutrition, both are emerging research interests in nutritional epidemiology. However, its association with glycemic control in the Asia population is less clear. A better understanding of how activity/eating time can influence glucose levels in Asian prediabetic individuals may improve strategies for blood glucose control in Asian countries. The present paper describes the research protocol which aims to determine the associations of chronotype and chrononutrition with glucose tolerance among Malaysian prediabetic individuals.

**Methods:**

This is a prospective longitudinal study named Chrono-DM™, that targets to recruit 166 newly diagnosed prediabetic individuals from the community clinics in Malacca, Malaysia. Respondents will be followed-up for 6 months: (1) baseline (1^st^ oral glucose tolerance test (OGTT)); (2) second visit (at 3^rd^ month); and (3) third visit (2^nd^ OGTT at 6^th^ month). Data collection includes sociodemographic and anthropometry measurements (weight, height, body fat, visceral fat, waist and hip circumference). Dietary intake and meal timing are collected using the 3-day dietary record while data on sleep pattern, light exposure, chronotype and chrononutrition will be collected using validated questionnaires. Physical activity will be recorded using a validated IPAQ questionnaire and pedometer during periods of using continuous glucose monitoring (CGM) sensor. CGM, fasting blood sugar (FBS), OGTT and HbA1c are performed to assess glycemic outcomes.

**Discussion:**

The Chrono-DM™ study represents a novel approach to determining the association of chronotype and chrononutrition with glycemic control. We anticipate that this study will not only review the association of chronotype with glycemia measure but also provide greater insight into optimal meal time for glycemic control among prediabetic individuals in the Asian population.

**Trial registration:**

NCT05163964 (Clinicaltrial.gov). Trial registration date:  20 December 2021.

**Supplementary Information:**

The online version contains supplementary material available at 10.1186/s12875-022-01815-7.

## Background

Diabetes mellitus burdens the healthcare system globally and regionally and is a leading cause of death in most countries [1]. Globally, an estimated 318 million people had prediabetes in 2015, with a projected alarming increase to 482 million in 2040 [2]. The prevalence of diabetes among Malaysian adults aged 18 years and above in Malaysia increased to 18.3% (3.9 million Malaysians) from 13.4% in 2015, indicating that 1 in 5 Malaysian adults have diabetes [3]. In general, an increasing trend was observed in undiagnosed hyperglycemia, from 5.1% in 2015 to 8.9% in 2019. Despite numerous strategies that have been implemented by the government and organizations, for example medical nutrition therapy; to the befuddlement of many diabetes researchers and clinicians, it has failed to substantially reduce the prevalence of T2DM from prediabetes globally. Perhaps, there is a missing component in the current medical nutrition therapy for diabetes.

Numerous studies have suggested that the main risk factors for T2DM are excessive caloric intake and a lack of exercise; which has been recommended in several diabetes management guidelines [1, 4, 5]. However, other factors such as chrononutrition alteration may also contribute to disease development [6]. Chrononutrition, an emerging nutrition epidemiology research, consists of several dimensions of eating behaviors, including timing, frequency, and regularity [7]. Unusual meal-timing will result in the shift of the circadian system, which may alter the activity of enzymes and thus, disturb hormone secretion, subsequently influencing the peripheral tissue metabolism and resulting in metabolic disorder [8]. In the past few years, the focus of nutrition research has shifted from nutrition composition to meal-timing, indicating the vital role of chrononutrition in metabolic health [9]. Perhaps, it is not what you eat but when you choose to eat that matters [10]. Recent evidence established that readjusted meal timing could reduce the risk of metabolic syndromes. However, no data is available on the transformation of these recommendations into routine care for patients with diabetes.

Additional epidemiology and experimental studies has suggested that sleep disorder is not only a risk factor for the onset of diabetes but also showed poor glycemic outcomes among type 2 diabetes [11]. Evening chronotypes typically have later bedtime habits than those who are morning type (early chronotype). If an individual shifts their biological clock by 2 to 3 h later at night, their internal circadian rhythms system desynchronizes with the period of the biological night, especially triggers by light exposure, leading to an increased risk of having type 2 diabetes [12]. Anothaisintawee et al*.* (2017) [13] also found that evening type prediabetic adults who have late bedtime behaviors are associated with higher HbA1c than morning-type prediabetic adults. Despite the role of the circadian system in regulating glucose hemostasis has been recognized, the relevant guidelines and information is not included in global medical nutrition therapy. Currently, most studies on circadian rhythm and circadian timing were conducted in western countries [14]. For example, Mezitis and Bhatnagar (2018) [15] from the United States of America (US) have recognized optimal hormonal and liver function beginning in the early morning (4:00 am) and fading in the early evening (4:00 pm). However, the times proposed by Mezitis and Bhatnagar (2018) [15] may only be suitable for diabetes patients in the United States and may not be able to apply to Asian diabetes management; as the circadian timing system are affected by factors consisting of cosmic events related to the universe and earth, environmental factors (light, night and day duration, seasons) and lifestyles.

Therefore, the purpose of our study is to develop an understanding of the role of chrononutrition and chronotype on blood glucose levels among prediabetic individuals, which may be modifiable behaviors for glycemic control. Our study namely Chrono-DM ™ where the chrono means relating to time and DM is the abbreviation of diabetes mellitus. To the best of our knowledge, this is the first study to explore the new concept of circadian timing in Asia (especially Malaysia) among prediabetic individuals. This proposed study aims (1) to examine the associations among chronotype, chrononutrition and glucose outcomes (2) to investigate the association between lifestyle factors (physical activity level, light exposure, diet timing, sleep pattern) with chrononutrition and (3) to investigate the association between anthropometry measurements and glycemic outcomes. We hypothesize that the morning chronotype and early meal time are associated with better glucose tolerance among prediabetic individuals.

## Methods/design

This is a prospective longitudinal observational study, which will be conducted from 2022 to 2024 using the convenience sampling method. The proposed study will be conducted in 11 community clinics in Central Malacca, Malaysia. The community clinics includes clinic Sungai Udang, clinic Bukit Rambai, clinic Batu Berendam, clinic Peringgit, clinic Seri Tanjung, clinic Tengkera, clinic Cheng, clinic Ayer Molek, clinic Ayer Keroh, clinic Ujong Pasir and clinic Klebang Besar. Malacca is the capital of a coastal state, located in the southwest of Malaysia, with an estimated total population of 0.93 million in 2019 [16]. In 2019, the National Health and Morbidity Survey reported that 17% of Malaccan adults had hyperglycemia and 13.7% of them had known diabetes. The Malacca state is selected because it is one of the top five states showing the highest prevalence of diabetes in Malaysia [3]. Data collection is started after obtaining ethical approval from Medical Research and permission to collect data from the Malacca Health Department of Malacca. The present study protocol is following SPIRIT guideline (Kindly refer to supplementary Table 1).

### Recruitment

Prediabetes and diabetes will be defined according to the latest Clinical Practice Guideline in Malaysia [5, 17, 18]. The diagnosis must be confirmed by measuring fasting venous blood sugar (FBS) and oral glucose tolerance test (OGTT) or the HbA1c level. Table 1 shows the diagnostic value of diabetes on plasma glucose. Table 2 shows the diagnostic values for prediabetes and diabetes based on OGTT. Table 3 shows the diagnostic values for prediabetes and diabetes based on HbA1c. All tables are adapted from the Clinical Practice Guideline [5, 17, 18]. The guideline also indicated that medical officers or family medicine specialist will refer newly diagnosed prediabetes individuals to dietitians and physiotherapists as the first line of a lifestyle intervention [5]. Dietitians will provide diet counseling and will inform potential prediabetic individuals of the research protocol verbally. Potential prediabetic individuals will be referred to a trained research assistant. In the following step, the trained research assistant identifies eligible prediabetic individuals and will invite them if they meet all the inclusion criteria as shown below:

1. Newly diagnosed prediabetic individuals who have first been seen by registered clinical dietitians under the Ministry of Health Malaysia.
2. Malaysian aged 18 to 69 years old.
3. Those who plan to continue prediabetes care at a community clinic in Malacca.
4. Those who can read, write and understand the Malay language.

**Table 1:** The diagnostic value for diabetes on plasma glucose

	Fasting	Random
Venous Plasma Glucose	≥ 7.0 mmol/L	≥ 11.1 mmol/L

In symptomatic individuals, an abnormal glucose value is diagnostic

In an asymptomatic individual, two abnormal glucose values are required

**Table 2:** The diagnostic values for prediabetes and diabetes based on OGTT

OGTT Plasma Glucose Values (mmol/L)		
Category	0 h	2- hour
Normal	< 6.1	< 7.8
IFG	6.1–6.9	-
IGT	-	7.8–11.0
DM	≥ 7.0	≥ 11.1

*IFG* Altered fasting glucose, *IGT* Impaired glucose tolerance, *DM* Diabetes mellitus

**Table 3:** The diagnostic values for prediabetes and diabetes based on HbA1c

	Normal	Pre-diabetes	Diabetes
HbA1c	< 5.6% (38 mmol/mol)	5.6–6.2% (38–44 mmol/mol)	≥ 6.3% (45 mmol/mol)

A repeated A1c should be done 4 weeks after the first positive test for asymptomatic patients

In symptomatic individuals, a single positive test is sufficient

Participants who meet the following criteria will be excluded from continuing the study.

1. Night shift workers (at least 8 h of work between 9.00 pm and 5.00am) at least 4 times a week.
2. Known sleep disorders (for example narcolepsy, sleep apnea, insomnia and others.)
3. Pregnant or lactating women.
4. Those on oral glucose-lowering medications, diabetes supplements, anticonvulsant medications or oral steroids currently or in the last month.
5. Those with prior information regarding the time of eating/time of activity restriction (for example practicing intermediate fasting to control their blood sugar level).
6. Those with chronic kidney disease. They are excluded as current research lack evidence to support the accuracy of using 24-h continuous glucose monitoring (CGM) system (Freestyle Libre Pro, Abbott, Germany).

During the recruitment process, the trained research assistant receives the study description and written informed consent from prediabetic individuals who agree to participate. Dietitians are witnesses to the process. The study description is a written information sheet that consists of the study procedure, data collection, data processing, data storage, name and contact information for researchers, potential risks, honorarium and possible withdrawal of consent. The contact list of researchers is also provided in the information sheet. The signed consent form is randomly assigned an ID number. Those who decline to participate will continue to receive their routine treatment as usual and care provided to each prediabetic individual. The patients care will not be affected by the decision to either participate or not participate in the study.

### Study endpoints

The main outcome of the study is the glycemic outcome including fasting blood sugar (FBS) (mmol/L), 2 h postprandial glucose (2hPPG) (mmol/L), and glycated hemoglobin (HbA1c) (%), and glucose variability from CGM sensor after follow-up assessment 6 months. Secondary outcomes are chronotype (morningness, intermediate and eveningness) from the validated Munich Chronotype questionnaire (MCTQ); chrononutrition such as meal timing, frequency, and regularity from the validated Malay translated chrononutrition profile questionnaire (CPQ-M); food qualities and quantities from 3 days dietary record (3DDR); height, weight, body mass index, waist and hip circumference from anthropometry measurements; body fat percentage, visceral fat and muscle mass from bioelectrical impedance analysis; physical activity level from the validated international physical activity questionnaire (IPAQ), sleep pattern from the validated questionnaires Pittsburgh Sleep Quality Index (PSIQ) and light exposure from the validated Harvard light exposure questionnaire (HLEQ) after 6 months follow-up.

### Data collection

Data is collected at baseline assessment, at the follow-up assessment 3 months and 6 months later based on the community clinic appointment schedules. In the baseline assessment, data regarding sociodemographic information will be obtained by the trained research assistant. Enrolled participants will be interviewed using the following questionnaires including (1) Munich Chronotype Questionnaires (MCTQ), (2) Pittsburgh Sleep Quality Index Questionnaire (PSQI), (3) Harvard Light Exposure Questionnaire (HLEQ), (4) International Physical Activity Questionnaire (IPAQ) and (5) Malay translated Chrononutrition Profile Questionnaire (CPQ-M). The anthropometry and body composition measurements, including weight, height, body fat percentage, visceral fat, waist circumference and hip circumference will be measured and recorded. Participants are requested to fill up 3-days dietary record (3DDR) (Two weekdays and one weekend day) and the data will be collected during the next visit. The 3DDR record progress will be followed up via phone. The baseline reading of fasting plasma glucose (FBS, OGTT, liver function, and HbA1c) will be retrieved from the medical records of the community clinic. Telephone conversations will be conducted to ensure that 3 days of dietary data are completed to minimize loss of follow-up.

At the 3-month follow-up assessment, all participants are required to wear a CGM sensor at the back of their upper arms to measure the 24 h glucose variability over 14 consecutive days. All participants are reminded to report any potential device-related, non-serious events related to continuous glucose monitor use to the trained research assistant during the period of wearing the CGM sensor. The trained research assistant will also inform the participants to remove CGM sensors if certain conditions as described in the section describing the risks are fulfilled. 3DDR will be given to participants to record their consecutive 3 days dietary records and will be returned after 14 days. The progress of the record will be followed up by phone. After 14 days, the participants will return the CGM sensor at the community clinic or will be collected by an assistant at the participant's home. All questionnaires will be again applied by a trained research assistant. Anthropometry and body composition measurements including weight, height, body fat percentage, visceral fat, muscle mass, waist circumference and hip circumference will be measured repeatedly. Fasting blood sugar and the HbA1c test will be collected from the patient’s medical records.

**Fig. 1:**
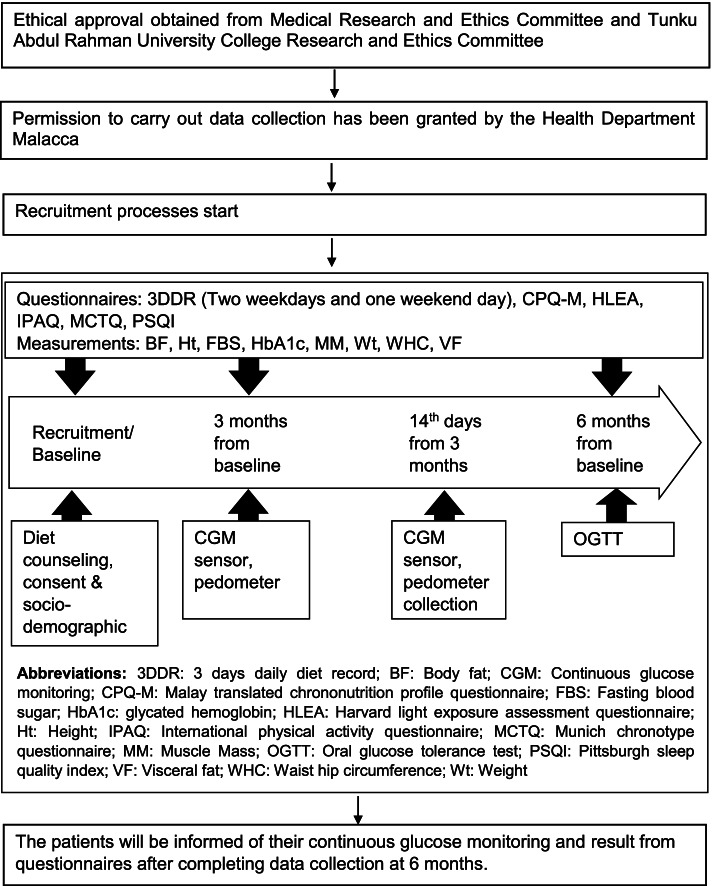
Flow chart of study design

At the 6-month follow-up evaluation, for FBS, OGTT and HbA1c tests, the research assistant will retrieve all the readings from the medical record to document the glycemic outcome. The participants will be interviewed by the trained research assistant during all clinic visits using the aforementioned questionnaires. All anthropometry and body composition measurements (weight, height, body fat percentage, visceral fat, muscle mass, waist circumference and hip circumference) will be conducted again. The 3DDR will be given to record their 3 days dietary intake again and the information will be obtained via phone. The original 3DDR will be collected at the routine appointment. The patient will be informed of his continuous glucose monitoring and the result of blood measurement after completing the data collection at 6 months. The final findings of the study will be distributed to participants upon their request by email. An overview of the study procedures is illustrated in Fig. 1.

### Study measurements

At the baseline assessment, sociodemographic information is collected based on questionnaires. The variables included age, sex (male, female), ethnicity (Chinese, Malay, Indian, others), education (no, primary, secondary, tertiary), occupation (employed, unemployed), household income, smoking status (Yes, former smoker, passive smoker, active smoker), medical status (recorded disease history), alcohol consumption (never, daily, weekly, monthly), lactating and pregnant (Yes, No), supplement intake (never, daily, weekly, monthly), exercise habits (never, daily, weekly, monthly) and known sleep disorder (Yes, No). Table 4 demonstrated the details of the variables collected at each stage of the study. The present study adopted all questionnaires either open access or permission obtained from the authors.

**Table 4:** Summary of data collection and timeline

Data	Baseline	3 months	6 months
**Sociodemographic**			
Age	**·**		
Sex	**·**		
Date of birth	**·**		
Education level	**·**		
Occupation	**·**		
Monthly household income	**·**		
Health history	**·**		
Alcohol habit			
Smoking habit	**·**		
Supplementary consumption	**·**		
Known sleep disorder	**·**		
Pregnant or lactating	**·**		
**Anthropometry**			
Weight	**·**	**·**	**·**
Height	**·**	**·**	**·**
Waist circumference	**·**	**·**	**·**
Visceral fat	**·**	**·**	**·**
Body fat	**·**	**·**	**·**
Step counts		**·**	
**Questionnaires**			
Munich chronotype (MCTQ)	**·**	**·**	**·**
Pittsburgh sleep quality index (PSQI)	**·**	**·**	**·**
Harvard light exposure (HLEQ)	**·**	**·**	**·**
International physical activity IPAQ)	**·**	**·**	**·**
Malay translated chrononutrition profile (CPQ-M)	**·**	**·**	**·**
3 days dietary record (3DDR)	**·**	**·**	**·**
**Glycemic Measurements**			
Continuous glucose monitoring (CGM)		**·**	
Fasting blood sugar (FBS)	**·**	**·**	**·**
2-h post-load plasma glucose (2hPPG)	**·**		**·**
Glycated hemoglobin (HbA1c)	**·**	**·**	**·**

### Glycemic measurements

In a community clinic, participants will receive routine laboratory tests, such as the FBS, OGTT, and HbA1c examination after 8 h of fasting. Firstly, a nurse will draw venous blood for FBS and HbA1c outcomes. This will be followed by giving 75 g glucose water within 5 min. Consequently, the blood sample will be taken after 2 h to determine 2hPPG. All blood samples will be analyzed in the community clinic on the same day. The FBS and 2hPPG samples will be analyze using the Dimension® clinical chemistry system (Siemens DF40, United States). The HbA1c samples will be analyzed using Hemoglobin A1c program (Bio-Rad® D-10, United States). The results will be collected and recorded by staff nurses. The blood samples will be kept in refrigerator 4 °C for 3 days and disposed accordingly. The value of fasting venous glucose and the result of OGTT will be documented in the medical file and recorded by the trained research assistant. The participants will be diagnosed with prediabetes if they have impaired glucose tolerance (2hPPG ≥ 7.8 mmol/L) and impaired fasting glucose (FBS 6.1–6.9 mmol/L) [5]. Prediabetes or diabetes conditions will be diagnosed by a family medicine specialist or medical officers. The family medicine specialist or medical officers will then refer the newly diagnosed prediabetic individual to a dietitian for diet counseling. Diet counseling will be scheduled for prediabetic individuals in the following days. The recruitment will begin after diet counseling. In the third month, participants are required to visit a community clinic for routine follow-up of FBS tests and HbA1c tests. The routine next appointment for prediabetic individuals in the community clinic is 6 months to measure the OGTT and HbA1c.

At the 3-month follow-up evaluation, a 24-h continuous glucose monitoring system will be used to measure the 24-h blood glucose variability for consecutive days. It is a wearable sensor that automatically detects the total glucose amount in interstitial fluids to determine the real-time glucose variability. Usage of a CGM sensor, typically Freestyle Libre Pro Flash Glucose Monitoring System (Abbott Diabetes Care Inc, Alameda, USA) is to assess the glucose fluctuation among prediabetic participants. All participants will be required to wear the CGM sensor at the back of the upper arm for a total of 14 consecutive days to monitor their glucose levels. The continuous glucose monitoring real-time reading will be kept anonymous to participants to avoid bias arising from unmasked real-time glucose reading. The sensor data will be accessed in the LibreView Cloud and downloaded for analysis.

### Meal timing, quality, and quantity

The 3 days dietary record (3DDR) is a common nutrition assessment tool applied to examine the quality, quantity, and meal eating pattern [19]. The tool is suitable for determining macronutrients and energy intake, especially meal patterns. At the first visit, a research assistant will brief 3DDR (2 weekdays and 1 weekend) for the baseline dietary intake data. The 3DDR will be returned to the research assistant during the following routine appointment. In the third month, participants will record their 3 days food and beverages intake again while wearing the CGM sensor throughout the 14 days. The diet record and the CGM sensor will be returned to the research assistant in the clinic during the next routine appointment. An information sheet explaining household measuring utensils will be given to participants to assist in the dietary food record. If participants missed the dietary records, a 24-h recall of dietary data will be conducted by telephone interview. In the 6^th^ month, the participants have to repeat the 3DDR. Participants will be contacted through phone to collect the dietary record and the original questionnaire will be collected at the next appointment.

Three days dietary record describes the typical diet daily intake and food consumption pattern, such information includes type and description of food and beverage taken throughout the days, brand information, ingredients, cooking method, and portion size based on household measuring utensils. Alongside, data on the timing of each main meal and snack will be recorded. Breakfast, lunch, and dinner are the main meal with 20–35% of total energy intake identified in the food daily whereas the rest like brunch, supper, and snacks (morning, afternoon tea, or beverage break) are the light meal taken in varied timing in between main meals [9]. Given Malaysians’ meal-timing varies individually, breakfast is the first meal taken early in the morning or until mid-morning (10.30 am) [20]. Lunch is the middle main meal between breakfast and dinner and is taken approximately between 12.30 pm to 2.30 pm [20]. Dinner is classified as the closing meal of the day which is consumed in the evening and night with an estimated time of 6.00 pm to 9.00 pm [20]. Night mealtime from 6.00 pm to 9.00 pm is considered common practice in Malaysia but different from the Malaysian Solar time zone where nighttime from 7.00 pm to 6.59 am based on circadian rhythms' responses to light. Using the 3DDR, data such as main and side dishes time, breakfast skipping, late-night eating patterns, and fasting behaviors will be obtained. Similarly, the variables like the number of eating occasions per day, night or midnight fasting duration, distribution of total energy intake in each meal, total eating window, and last meal of the day will be extracted through the nutrition assessment tool.

With the record of food type and total food amount in grams, the distribution of total energy and calorie intake will be tabulated. The collected food database will be analyzed using Nutritionist Pro™ (Axxya Systems, Stafford, TX, USA) which is comprised of the Malaysian Food Composition Database [21]. Total energy intake (TEI) will be compared with the estimated basal metabolic rate to evaluate whether there is underreporting or an overreporting of their results. Meanwhile, the basal metabolic rate (BMR) of Malaysian adults will be calculated using the formula of Ismail et al*.* (1998) [22]. According to Goldberg et al*.*, (1991) [23] and Black, (2000) [24], the ratio between TEI and BMR is 1.2 and 2.4, or else, it will be considered underreported if less than 1.2 and overreported if more than 2.4. All the under and over-reporting dietary data will be excluded from the data analyses. Although the 3-day diet record is used to assess the food diary, the compliance and adherence of the respondents on the 3-day dietary record limits the report of their actual meal timing. On that account, the trained research assistants are required to follow up on their records through phone conversations.

### Anthropometry measurements and body composition

The outcomes will be assessed on three occasions, at baseline, at 3 months and at 6 months. All assessments will be completed by the site principal investigator to avoid inter-interviewer variations. Participants’ weight (Omron Karada Scan HBF-375, Kyoto, Japan) and height (SECA 213 GmbH & Co., Hamburg, Germany) will be measured at least twice according to the standard protocol to obtain their body mass index [25]. During weight measurement, minimum clothing on, all pocket items and jewelry will be removed, and the measurement will be recorded to the nearest 0.1 kg. Height measurements will be requested, and participants must stand straight with bare feet, aligned with heel, buttocks, head, and shoulder in a vertical line against the wall. Approximately nearest 0.1 cm height reading is taken with twice repeated measurements. The category of body mass index based on WHO, (1997) [26], which indicated < 18.5 kg/m^2^ is low body weight, 18.5–24.9 kg/m^2^ is normal, and ≥ 25 kg/m^2^ is overweight and obese.

The body composition monitor model (Omron Karada Scan HBF-375, Kyoto, Japan) is used to measure weight, body fat, visceral fat level, body age, and resting metabolism. The reading of different areas of subcutaneous fat and skeletal muscle will be documented [27]. To reduce erroneous results during body composition monitoring, participants have to remove all metal-related items before weighing electrodes of the scale will be cleaned to remove any debris. Waist circumference will be measured around the midpoint between the lowest margin of the lowest palpable rib and the top of the iliac crest using Lufkin tape (Lufkin® Executive Thinline W606 Apex Tool Group, Dallas, TX, USA), and the reading nearest to 0.1 cm will be recorded and repeated twice. The average values will be taken from two readings of all anthropometry measurements in the final report.

### Chrononutrition

The chrononutrition profile questionnaire (CPQ) is a validated tool to assess the overall trend of chrononutrition and their practice in a typical week, neither 5 workdays nor 2 free days in the general population [28]. The questionnaire is translated into Malay and validated to ensure cross-cultural adaptation, especially Malaysians [29]. It consists of six different chrononutrition behaviors, which are breakfast skipping, largest meal, evening eating, evening latency, nighttime eating, and eating window. Similarly, this questionnaire provides some eating pattern information similar to 3DDR, but it incorporates sleep–wake habitual and eating misalignment (difference between eating time preference and real practice) [28]. Scoring guidance is provided for each item to calculate the discrepancy between workday and free day values. The sleep timing variables, the eating timing variables, the weekly average eating window, and the meal frequency variables will be obtained from the score calculation. The participants will be interviewed the questionnaire by a research assistant every 3 visits.

### Chronotype

Chronotype is defined as the preferred time of a day to perform their personal daily sleep–wake activities within 24 h based on their circadian rhythm [30]. Munich Chronotype Questionnaire (MCTQ) is the first questionnaire developed by Roenneberg et al. (2003) [31] to determine the chronotype in the adult population. Mazri et al*.* (2021) [32] modified, validated and translated the Munich chronotype questionnaire in the Malay language and applied it to assess the chronotype among Malaysians. This is because religious beliefs and morning prayers among Malaysians demonstrated the disturbance of sleep at mid-time despite work or free days. We adopted this questionnaire in our study to control covariates in responding to our primary outcome. This questionnaire consists of a total of 21 items and will be interviewed by a research assistant during every visit.

### Physical activity, sedentary lifestyle, sleep and light exposure

Lifestyle factors such as physical activity, sedentary lifestyle, sleep quality, and light exposure will be collected as a record to adjust the covariates that responded to the primary outcome. Pedometer Digi-walker CW-701 (Yamax, Fukuyama, Japan) will be used to calculate the real-time step counting among prediabetic participants during the CGM sensor monitoring period. 10,000 steps are generally recommended for adult populations to encourage them to be physically active daily. Step counts classification as sedentary lifestyle refers to adults with less than 5000 steps per day; physically inactive with 5000 to 7499 steps daily, meanwhile, 7500 to 9999 steps daily are moderately active. If adults achieve more than 12,500 steps per day, they will be labeled as very active lifestyle behavior [33]. In this study, participants are trained to wear the pedometer for the first 7 days of the CGM sensor monitoring period to track their daily steps and will be return together with the CGM sensor on day 14^th^. The open access International physical activity questionnaire short form (IPAQ-short) adopted by validated Malay version in Malaysian adults population from Soo et al. 2015 [34] to determine the physical activity level past 7 days among prediabetic participants in this study. The data from IPAQ-M will be computerized in metabolic equivalents score (MET/min/week) and its calculation based on Craig et al. 2003 [35]. The level of physical activity has been categorized into three different levels, which are inactive, minimally active, and highly active. The definition of each category is found in the scoring of Craig et al. 2003 [35]. Sedentary lifestyle items included a questionnaire to assess sedentary behavior which items are adopted from the adult Sedentary Behavior Questionnaire.

Studies have suggested that circadian rhythms that initialize the production of the hormone melatonin regulate our sleep–wake system [36]. Social jetlag triggers sleep disorders by affecting the regulation of the hormone melatonin, consequently, leading to poor glycemic outcomes [37]. Given Pittsburgh Sleep Quality Index Questionnaire (PSQI) which is validated in Malay populations, the sleep pattern over a month shall be assessed through interviewed-administrative [38, 39]. Sleep quality, latency, duration, efficiency, disturbances, medication use, and daytime dysfunction will generate a score to differentiate between good and poor sleep disorder problems [38]. Alongside, another tool namely Harvard Light Exposure Questionnaire will be applied to determine the various light source explored hourly on work and free days [40]. This tool is suitable for understanding light exposure among prediabetic individuals and identifying its relationship to glycemic outcomes.

### Sample size calculation

The sample size is estimated using G*Power software version 3.1.9.4 [41]. Given that late-night dinner was associated with HbA1c after adjusted potential covariates (standardized β-coefficient: 0.13, *p* = 0.028) adopted by Sakai et al*.* (2018) [42], we aimed for a sample size of 83 adults with prediabetes that provide us with a minimum power of 90% with a two-sided significance level and an alpha error given 0.05. To compensate for the non-responding and non-compliance during follow-up, a dropout rate of 50% will be considered, the total sample size required is 166 subjects in the study for 6 months.

### Data protection

All participants will receive a printed information sheet during the baseline recruitment. The data of the consent form will be kept confidential in electronic form and secured with password protection by a trained research assistant. All data evaluated in the baseline and follow-up assessment will be pseudonymously documented. The trained research assistant will enter the data of questionnaires and consent form and a trained registered dietitian will complete the data entry of 3 days dietary records in electronic forms. The consent forms, CGM sensors and questionnaires in both hard copies and electronic forms will be stored in the Tunku Abdul Rahman University College cabinet at least three years [43]. They will be disposed of for safety after 3 years.

### Quality Control

To ensure that the database accurately reflects the data reported in the questionnaires, a pilot test will be carried out. The research assistant will be trained to carry out the study procedures including obtaining administrative questionnaires, CGM device, 3 days dietary record, and pedometer. A monthly report will be documented after the meeting. A weekly recruitment status will be reported to all co-researchers and supports provided for the recruitment process.

### Description of risks

The CGM system (Freestyle Libre System Pro IQ) is a continuous glucose monitoring system approved by the US Food and Drug Administration (No: K212132) and registered with Malaysia Medical Device Authority (Registration No: RSCH ID-21–00,114-IVI). The Malaysia Medical Device Authority approved CGM system (Freestyle Libre System Pro IQ) to be use in current study. In the present study, this system will be used fully to observe the personality of glucose variability, but not with the purpose to diagnose hyperglycemia or hypoglycemia. The Freestyle Libre System Pro IQ must be removed prior to magnetic resonance imaging (MRI), Computed Tomography (CT) scan, or high-frequency electrical heat (diathermy) treatment [44]. Potential device-related, non-serious events related to continuous glucose monitor use includes local infection, skin irritation or redness, skin inflammation, pain or discomfort, bleeding, bruising, skin edema, skin rash, itching, scarring or skin discoloration, allergic reactions to the sensor needle or adhesives, sensor or needle fracture during insertion, wear or removal. Sensor breakage with fragments retained under the skin is a potential procedure-related complication. However, based on post-market experience with this and similar devices, and the results observed in the clinical study, this event is rare and its severity does not raise major concerns. Participation in this study is of minimal risk. The medical officers and family medicine specialist will monitor the adverse events of wearing CGM sensors and will provide the clinical support if necessary. However, participants are free to stop at any time during data collection if they feel uncomfortable.

### Ethics approval

A written information sheet about the study protocol will be provided to participants and written consent is obtained during recruitment. Any changes in the study protocol or instruments used shall obtain approval from the ethical committee, followed by a re-consent of the participants whenever necessary. The ethical approval has been obtained from the Medical Research and Ethics Committee (NMRR ID-21–02,090-SKX (IIR)) and the Tunku Abdul Rahman University College Ethics Committee (TAR UC/EC/2021/02–3). The findings of the study will be presented at a conference and disseminated in peer-reviewed journals.

### Data analysis

Data profiling and cleansing will be performed by a trained research assistant to assess the data quality and correct various structural errors in datasets, such as misspellings and other typographical errors, wrong numerical entries, syntax errors and missing values. The trained registered dietitian will verify the overall datasets to ensure the overall cleanliness and avoid inconsistent data, duplicate data and irrelevant data appearing in the final datasets. Statistical analysis using the SPSS software version 20 (SPSS Inc., Chicago, IL, USA) will be performed by the trained research assistant. Sociodemographic data will be presented as mean, standard deviations, median, interquartile range, frequency, and percentage. Data cleaning will be performed to exclude the outlier data before the normality is checked using the Kolmogorov–Smirnov test. This study considers *p* < 0.05 to be statistically significant. Chronotype, sleep quality, and light exposure will be analyzed as categorical variables while dietary intake, chrononutrition, anthropometry, glycemic measure and physical activity will be analyzed as continuous variables.

We will perform a multivariate generalized linear model to examine the association between night meal pattern (last night mealtime (dinner or supper) with calories intake, frequency of night snacking, and the total number of the night snacking) and glycemic outcome (HbA1c, FBS, 2hPPG, CGM profile) by adjusting for potential covariates (sociodemographic characteristics, light exposure, diet quality, physical activity, and sleep pattern). Specifically, nighttime will be characterized by a time range from 7.00 pm and 6.59 am based on Malaysia solar time. We will explore the timing at 8 pm, 9.00 pm, and 10.00 pm to examine its association with glycemic outcomes. Potential confounders include sociodemographic characteristics, physical activity, sleep pattern, sedentary behavior, diet quality, and light exposure will be controlled. The ANCOVA test will be performed to understand the association between chrononutrition (breakfast skipping, meal frequency, eating window, sleep timing, chronotype, and fasting pattern) and glycemic variables, controlling for potential covariates (sociodemographic characteristics, light exposure, diet quality, physical activity, and sleep pattern).

To investigate the association of physical activity (MET score and steps count), sedentary behaviors (MET score), sleep pattern (sleep duration), diet quality (distribution of total energy, carbohydrate, protein and fat), and light exposure (timing responded to a different type of light) with the glycemic result, the multivariable generalized linear model will be performed. Meanwhile, anthropometric measures (body mass index, body fat, and waist circumference) will be categorized as either normal or overweight and obesity group, followed by using logistic regression to examine the association between anthropometry status and glycemic outcome (non-diabetes or diabetes). Additionally, the glycemic outcome will be classified into diabetes incidence (diabetes and non-diabetes) to determine participants who had been diagnosed with diabetes at 6 months. A logistic regression test will be used to examine the association between early and late-night mealtime and diabetes incidence.

## Discussion

Prediabetic individuals are those with intermediate hyperglycemic and their treatment is similar to that of patients with type 2 diabetes and have the potential to reverse their high blood glucose level with a proper lifestyle intervention [45]. The discovery of the circadian rhythm in glucose homeostasis and its disruption by feeding patterns illustrated the concept of chrononutrition and chronotype, which are important in the management of diabetes. To compose a biological clock, sleep pattern, and nutrition, a new concept of chronotype and chrononutrition is utilized in current research, to determine the role of activity/ feeding timing in disease prevention. To the best of our knowledge, this is the first study that aims to determine the association between chrononutrition and chronotype with glucose tolerance among prediabetic individuals in Asia.

### Expected implications for practice

According to currently available data, medical nutrition therapy for Malaysian diabetic patients should include meal timing as one of the strategies to manage glycemic control. A study should be conducted to determine the optimal meal timing to manage blood glucose levels, as well as to determine the associations between chronotype and chrononutrition with glucose tolerance. Although it is very difficult to control meal timing for every individual including prediabetic adults, a socio-economical approach such as recommendations regarding the work environment may also be important. There is now insufficient evidence to evaluate the impact of these chrono-physiological disruptions on the progression or pathophysiology of diabetes precisely, particularly in Asia. Future-focused research on these issues can answer these questions.

### Expected implications for research

The strength of this study includes the use of a continuous glucose monitoring system to investigate the glucose variability which will reflect the glucose spike caused by real-time meal consumption and cross-check with 3 days dietary records. The dietary record method is more reliable instead of the memory-replying recall method. To control the covariate, physical activity level is determined using a pedometer to collect the real-time step count and followed by compared with 7 days physical activity recall using the validated International Physical Activity Questionnaire. A validated light exposure questionnaire is used to identify the duration of individuals exposed to different types of light as the covariate control. The chrononutrition profile questionnaire also strengthens the eating misalignment with circadian rhythms. This is the first study performed in Asia, with a novel approach to feeding behavior observation among prediabetes.

The proposed study may be limited by its external validity as it only includes prediabetic individuals from one state in Malaysia. Convenience sampling can result in selection bias due to the participants’ recruitment restrictions of participants at the study site. The selection will strictly follow the inclusion and exclusion criteria to ensure the outcome. Multiple questionnaires used during assessment may reduce the adherence and compliance of participants, thereby the participant will be interviewed by a research assistant and a travel allowance given after completing the data collection. This study has the potential to be conducted in a clinical trial to determine the application of activity/meal timing and its association with glucose tolerance or the more established circadian rhythm.

### Expected implications for health policy

Identifying novel, potentially modifiable, chronotype, chrononutrition, and lifestyle factors associated with glycemic measures can lead to innovative strategies to improve health status and prevent T2DM in prediabetic individuals. The proposed study presents a novel approach as a paradigm shift in Malaysia diabetes management. We anticipate that this study will not only reveal the optimal meal feeding for Malaysian prediabetic individuals to improve their glucose tolerance, but it will also provide greater insight into the associations of chronotype, chrononutrition, lifestyle factors and anthropometric measurements with glycemic measures. The findings will also set the directions to adopt the optimal-meal-timing approach in the Clinical Practice Guideline for diabetes. We anticipate that our proposed study which incorporates circadian timing, as a novel approach, may provide fundamental information that could be adopted by the government and policymakers. It can strengthen our existing treatment strategies via medical diabetic nutrition so that we can achieve a reduction of 15% prevalence of diabetes in 2025 according to Malaysia National Strategy Plan for Non-Communication Disease [46]. The outcome can be included in the future clinical practice guideline or to develop innovative and effective lifestyle interventions to improve the T2DM prevalent in Malaysia. In addition, this study will provide methodological support for the efficient planning of further interventional research.

## Supplementary Information

**Additional file 1: Table S1.** SPIRIT 2013 checklist for study Chrono-DM^TM^.

## Data Availability

Data sharing is not applicable to these articles as no datasets were generated or analyzed during the current study.

## Abbreviations

2hPPG: 2-Hour postprandial glucose
3DDR: 3 Days of diet record
BMI: Body mass index
CGM: Continuous glucose monitoring
CPQ: Chrononutrition profile questionnaire
CPQ-M: Malay translated chrononutrition profile questionnaire
DM: Diabetes mellitus
FBS: Fasting blood sugar
HbA1c: Glycated hemoglobin
HLEQ: Harvard light exposure questionnaire
IFG: Impaired fasting glucose
IGT: Impaired glucose tolerance
IPAQ: International physical activity questionnaire
MCTQ: Munich chronotype questionnaires
OGTT: Oral glucose tolerance test
PSQI: Pittsburgh sleep quality index questionnaire
