# Supplementary material for: Chronotype, chrononutrition and glucose tolerance among prediabetic individuals: research protocol for a prospective longitudinal study Chrono-DM™
Source: BMC Prim Care. 2022 Aug 4;23:193. doi: 10.1186/s12875-022-01815-7 (PMC9351220; doi:10.1186/s12875-022-01815-7)
Supplement: Supplementary file 1 — Additional file 1: Table S1. SPIRIT 2013 checklist for study Chrono-DMTM. [file 12875_2022_1815_MOESM1_ESM.docx]

Supplementary Table 1: SPIRIT 2013 checklist for study Chrono-DM^TM^.

| Section/item | Item No | Description | Sections/ Pages/  Lines | Contents |
| --- | --- | --- | --- | --- |
| **Administrative information** | | |  |  |
| Title | 1 | Descriptive title identifying the study design, population, interventions, and, if applicable, trial acronym | Page 1, Lines 1 to 2. | Chronotype, chrononutrition and glucose tolerance among prediabetic individuals: research protocol for a prospective longitudinal study Chrono-DM^TM^. |
| Trial registration | 2a | Trial identifier and registry name. If not yet registered, name of intended registry | Section Abstract, page 2, line 52 50 53 | Trial registration number: NCT05163964 (Clinicaltrial.gov)  Trial registration date: 29 June 2022 |
|  | 2b | All items from the World Health Organization Trial Registration Data Set | Not applicable | Not applicable |
| Protocol version | 3 | Date and version identifier | Page 1, Line 25. | Protocol version 02, 21 July 2022 |
| Funding | 4 | Sources and types of financial, material, and other support | Page 18, Lines 652 to 654. | The research is funded by the Ministry of Higher Education Malaysia, Fundamental Research Grant Schema (FRGS/1/2021/SKK06/TARUC/02/1) and HCK received the fund. |
| Roles and responsibilities | 5a | Names, affiliations, and roles of protocol contributors | Page 1, lines 4 to 18  Section Declarations, page 18, lines 659 to 663. | Guey Yong Chong^1^… ^7^Hospital Serdang, Selangor, Malaysia.  HCK, SK, RAT, SLL, WYS, HYT, RBA, HBM, and SSHS led the conceptualization and design of the study. GYC drafted the manuscripts. HCK, SK, RAT, SLL, WYS and HYT substantially revised the manuscript. WYS, RBA, HBM and SSHS will provide clinical expertise. GYC, HCK and SK will perform data analysis and interpretation. All authors have read, reviewed and approved the final manuscript. |
|  | 5b | Name and contact information for the trial sponsor | Section Declarations, page 18, Lines 652 to 654.  Section Recruitment, page 6, lines 177 to 180. | The research is funded by the Ministry of Higher Education Malaysia, Fundamental Research Grant Schema (FRGS/1/2021/SKK06/TARUC/02/1) and HCK received the fund.  The study description is a written information sheet that consists of the study procedure, data collection, data processing, data storage, name and contact information for researchers, potential risks, honorarium and possible withdrawal of consent. |
|  | 5c | Role of study sponsor and funders, if any, in study design; collection, management, analysis, and interpretation of data; writing of the report; and the decision to submit the report for publication, including whether they will have ultimate authority over any of these activities | Section Declarations, page 18, lines 654 to 656. | The funders had no role in the study design; collection, management, analysis and interpretation of data; preparation of the manuscript decision, or submission of the report for publication. |
|  | 5d | Composition, roles, and responsibilities of the coordinating centre, steering committee, endpoint adjudication committee, data management team, and other individuals or groups overseeing the trial, if applicable (see Item 21a for data monitoring committee) | Not applicable | Not applicable |
| Introduction | | |  |  |
| Background and rationale | 6a | Description of research question and justification for undertaking the trial, including summary of relevant studies (published and unpublished) examining benefits and harms for each intervention | Section Background, pages 3 to 4, Lines 60 to 114. | Diabetes mellitus burdens the healthcare system globally and regionally and is a leading cause of death in most countries … In the past few years, the focus of nutrition research has shifted from nutrition composition to meal-timing, indicating the vital role of chrononutrition in metabolic health (St-Onge *et al.*, 2017). Perhaps, it is not what you eat but when you choose to eat that matters (Jiang and Turek, 2017). Recent evidence established that readjusted meal timing could reduce the risk of metabolic syndromes. However, no data is available on the transformation of these recommendations into routine care for patients with diabetes…. Despite the role of the circadian system in regulating glucose hemostasis has been recognized, the relevant guidelines and information is not included in global medical nutrition therapy. Currently, most studies on circadian rhythm and circadian timing were conducted in western countries…However, the times proposed by Mezitis and Bhatnagar (2018) may only be suitable for diabetes patients in the United States and may not be able to apply to Asian diabetes management … Our study namely Chrono-DM ^TM^ where the chrono means relating to time and DM is abbreviation of diabetes mellitus. |
|  | 6b | Explanation for choice of comparators | Not applicable | Not applicable |
| Objectives | 7 | Specific objectives or hypotheses | Section Background, Page 4, Lines 115 to 122 | This proposed study aims (1) to examine the associations among chronotype, chrononutrition and glucose outcomes (2) to investigate the association between lifestyle factors (physical activity level, light exposure, diet timing, sleep pattern) with chrononutrition and (3) to investigate the association between anthropometry measurements and glycemic outcomes. The central hypothesis is that the morning chronotype and early meal time are associated with better glucose tolerance among prediabetic individuals. |
| Trial design | 8 | Description of trial design including type of trial (eg, parallel group, crossover, factorial, single group), allocation ratio, and framework (eg, superiority, equivalence, noninferiority, exploratory) | Section Methods/Design, Page 5, Lines 124 to 125.  Page 22, Lines 812 to 872, Figure 1. | This is a prospective longitudinal observational study, which will be conducted from 2022 to 2024 using the convenience sampling method.  The flow chart of the study procedure is described in Figure 1. |
| Methods: Participants, interventions, and outcomes | | | | |
| Study setting | 9 | Description of study settings (eg, community clinic, academic hospital) and list of countries where data will be collected. Reference to where list of study sites can be obtained | Section Methods/Design, Page 5, Lines 125 to 129. | The proposed study will be conducted in 11 community clinics in Central Malacca, Malaysia. The community clinics includes clinic Sungai Udang, clinic Bukit Rambai, clinic Batu Berendam, clinic Peringgit, clinic Seri Tanjung, clinic Tengkera, clinic Cheng, clinic Ayer Molek, clinic Ayer Keroh, clinic Ujong Pasir and clinic Klebang Besar. |
| Eligibility criteria | 10 | Inclusion and exclusion criteria for participants. If applicable, eligibility criteria for study centres and individuals who will perform the interventions (eg, surgeons, psychotherapists) | Section recruitment, pages 5 to 6, lines 151 to 173. | In the following step, the trained research assistant identifies eligible prediabetic individuals and will have invited...  1) Newly diagnosed prediabetic individuals who have first been seen by registered clinical dietitians under the Ministry of Health Malaysia.  2) Malaysian aged 18 to 69 years old.  3) Those who plan to continue prediabetes care at a community clinic in Malacca.  4) Those who can read, write and understand the Malay language.  Participants who meet the following criteria will be excluded from continuing the study.  1) Night shift workers (at least 8 hours of work between 9.00pm and 5.00am) at least 4 times a week.  2) Known sleep disorders (for example narcolepsy, sleep apnea, insomnia and others.)  3) Pregnant or lactating women.  4) Those on oral glucose-lowering medications, diabetes supplements, anticonvulsant medications or oral steroids currently or in the last month.  5) Those with prior information regarding the time of eating/time of activity restriction (for example practicing intermediate fasting to control their blood sugar level).  6) Those with chronic kidney disease. They are excluded as current research lack evidence to support the accuracy of using 24-hour continuous glucose monitoring (CGM) system (Freestyle Libre Pro, Abbott, Germany).  … using a 24-hour continuous glucose monitoring system (CGMS) (Freestyle Libre Pro, Abbott, Germany). |
| Interventions | 11a | Interventions for each group with sufficient detail to allow replication, including how and when they will be administered | Not applicable | Not applicable |
|  | 11b | Criteria for discontinuing or modifying allocated interventions for a given trial participant (eg, drug dose change in response to harms, participant request, or improving/worsening disease) | Not applicable | Not applicable |
|  | 11c | Strategies to improve adherence to intervention protocols, and any procedures for monitoring adherence (eg, drug tablet return, laboratory tests) | Not applicable | Not applicable |
|  | 11d | Relevant concomitant care and interventions that are permitted or prohibited during the trial | Not applicable | Not applicable |
| Outcomes | 12 | Primary, secondary, and other outcomes, including the specific measurement variable (eg, systolic blood pressure), analysis metric (eg, change from baseline, final value, time to event), method of aggregation (eg, median, proportion), and time point for each outcome. Explanation of the clinical relevance of chosen efficacy and harm outcomes is strongly recommended | Section study endpoints, Page 6, Lines 187 to 200. | The main outcome of the study is the glycemic outcome including fasting blood sugar (FBS) (mmol/L), 2 hours postprandial glucose (2hPPG) (mmol/L), and glycated hemoglobin (HbA1c) (%), and glucose variability from CGM sensor after follow-up assessment 6 months. Secondary outcomes are chronotype (morningness, intermediate and eveningness) from the validated Munich Chronotype questionnaire (MCTQ); chrononutrition such as meal timing, frequency, and regularity from the validated Malay translated chrononutrition profile questionnaire (CPQ-M); food qualities and quantities from 3 days dietary record (3DDR); height, weight, body mass index, waist and hip circumference from anthropometry measurements; body fat percentage, visceral fat and muscle mass from bioelectrical impedance analysis; physical activity level from the validated international physical activity questionnaire (IPAQ), sleep pattern from the validated questionnaires Pittsburgh Sleep Quality Index (PSIQ) and light exposure from the validated Harvard light exposure questionnaire (HLEQ) after 6 months follow-up. |
| Participant timeline | 13 | Time schedule of enrolment, interventions (including any run-ins and washouts), assessments, and visits for participants. A schematic diagram is highly recommended (see Figure) | Section Methods/Design, Page 22, Lines 812 to 872, Figure 1. | We have described the flow chart of the study procedure in Figure 1. |
| Sample size | 14 | Estimated number of participants needed to achieve study objectives and how it was determined, including clinical and statistical assumptions supporting any sample size calculations | Section Sample Size Calculation, pages 11 to 12, Lines 441 to 448. | The sample size is estimated using G*Power software version 3.1.9.4 (Erdfelder, Faul and Buchner, 1996). Given that late-night dinner was associated with HbA1c after adjusted potential covariates (standardized β-coefficient: 0.13, p=0.028) adopted by Sakai *et al.* (2018), we aimed for a sample size of 83 adults with prediabetes that provide us with a minimum power of 90% with a two-sided significance level and an alpha error given 0.05. To compensate for the non-responding and non-compliance during follow-up, a dropout rate of 50% will be considered, the total sample size required is 166 subjects in the study for 6 months. |
| Recruitment | 15 | Strategies for achieving adequate participant enrollment to reach target sample size | Section recruitment, page 5, Lines 146 to 151. | The guideline also indicated that medical officers or family medicine specialist will refer newly diagnosed prediabetes individuals to dietitians and physiotherapists as the first line of a lifestyle intervention (MOH *et al.*, 2020)^[[1]](#footnote-1)^. Dietitians provide diet counseling and will inform potential prediabetic individuals of the research protocol verbally. Potential prediabetic individuals are referred to a trained research assistant. |
| **Methods: Assignment of interventions (for controlled trials)** | | | | |
| Allocation: | | |  |  |
| Sequence generation | 16a | Method of generating the allocation sequence (e.g., computer-generated random numbers), and list of any factors for stratification. To reduce predictability of a random sequence, details of any planned restriction (e.g. blocking) should be provided in a separate document that is unavailable to those who enroll participants or assign interventions | Not applicable | Not applicable |
| Allocation concealment mechanism | 16b | Mechanism of implementing the allocation sequence (eg, central telephone; sequentially numbered, opaque, sealed envelopes), describing any steps to conceal the sequence until interventions are assigned | Not applicable | Not applicable |
| Implementation | 16c | Who will generate the allocation sequence, who will enrol participants, and who will assign participants to interventions | Not applicable | Not applicable |
| Blinding (masking) | 17a | Who will be blinded after assignment to interventions (eg, trial participants, care providers, outcome assessors, data analysts), and how | Not applicable | Not applicable |
|  | 17b | If blinded, circumstances under which unblinding is permissible, and procedure for revealing a participant’s allocated intervention during the trial | Not applicable | Not applicable |
| **Methods: Data collection, management, and analysis** | | | | |
| Data collection methods | 18a | Plans for assessment and collection of outcome, baseline, and other trial data, including any related processes to promote data quality (eg, duplicate measurements, training of assessors) and a description of study instruments (eg, questionnaires, laboratory tests) along with their reliability and validity, if known. Reference to where data collection forms can be found, if not in the protocol | Section Methods/Design, Pages 6 to 12, Lines 203 to 438. | Data is collected at baseline assessment, at the follow-up assessment 3 months and 6 months later … Enrolled participants will be interviewed using the following questionnaires including (1) Munich Chronotype Questionnaires (MCTQ), (2) Pittsburgh Sleep Quality Index Questionnaire (PSQI), (3) Harvard Light Exposure Questionnaire (HLEQ), (4) International Physical Activity Questionnaire (IPAQ) and (5) Malay translated Chrononutrition Profile Questionnaire (CPQ-M) … At the baseline assessment, sociodemographic information is collected based on questionnaires … The FBS and 2hPPG samples will be analyze using the Dimension^®^ clinical chemistry system (Siemens DF40, United States). The HbA1c samples will be analyzed using Hemoglobin A1c program (Bio-Rad^®^ D-10, United States)… The 3 days dietary record (3DDR) is a common nutrition assessment tool applied to examine the quality, quantity, and meal eating pattern…. Participants’ weight (Omron Karada Scan HBF-375, Kyoto, Japan) and height (SECA 213 GmbH & Co., Hamburg, Germany) will be measured at least twice according to the standard protocol to obtain their body mass index… The chrononutrition profile questionnaire (CPQ) is a validated tool to assess the overall trend of chrononutrition… Munich Chronotype Questionnaire (MCTQ) is the first questionnaire… to determine the chronotype in the adult population... Pedometer Digi-walker CW-701 (Yamax, Fukuyama, Japan) will be used to calculate the real-time step counting among prediabetic participants during the CGM sensor monitoring period… Given Pittsburgh Sleep Quality Index Questionnaire which is validated in Malay populations, the sleep pattern over a month shall be assessed through interviewed-administrative… Harvard Light Exposure Questionnaire will be applied to determine the various light source explored hourly on work and free days… identifying its relationship to glycemic outcomes. |
|  | 18b | Plans to promote participant retention and complete follow-up, including list of any outcome data to be collected for participants who discontinue or deviate from intervention protocols | Section Methods/Design, Page 24, Line 909, Table 4. | The summary of data collection and timeline have described in Table 4. |
| Data management | 19 | Plans for data entry, coding, security, and storage, including any related processes to promote data quality (eg, double data entry; range checks for data values). Reference to where details of data management procedures can be found, if not in the protocol | Section data protection, Page 12, Lines 452 to 459.  Section data analysis, Page 13, Lines 502 to 507. | The data of the consent form will be kept confidential in electronic form and secured with password protection by a trained research assistant. All data evaluated in the baseline and follow-up assessment will be pseudonymously documented. The trained research assistant will enter the data of questionnaires and consent form and a trained registered dietitian will complete the data entry of 3 days dietary records in electronic forms. The consent forms, CGM sensors and questionnaires in both hard copies and electronic forms will be stored in the Tunku Abdul Rahman University College cabinet at least three years (Coulehan and Wells, 2012). They will be disposed of for safety after 3 years.  Data profiling and cleansing will be performed by a trained research assistant to assess the data quality and correct various structural errors in datasets, such as misspellings and other typographical errors, wrong numerical entries, syntax errors and missing values. The trained registered dietitian will verify the overall datasets to ensure the overall cleanliness and avoid inconsistent data, duplicate data and irrelevant data appearing in the final datasets. |
| Statistical methods | 20a | Statistical methods for analysing primary and secondary outcomes. Reference to where other details of the statistical analysis plan can be found, if not in the protocol | Section data analysis, pages 13 to 14, lines 507 to 544. | Statistical analysis using the SPSS software version 20 …We will perform a multivariate generalized linear model to examine the association between night meal pattern (last night mealtime (dinner or supper) with calories intake, frequency of night snacking, and the total number of the night snacking) and glycemic outcome (HbA1c, FBS, 2hPPG, CGM profile) by adjusting for potential covariates (sociodemographic characteristics, light exposure, diet quality, physical activity, and sleep pattern)… The ANCOVA test will be performed to understand the association between chrononutrition (breakfast skipping, meal frequency, eating window, sleep timing, chronotype, and fasting pattern) and glycemic variables, controlling for potential covariates (sociodemographic characteristics, light exposure, diet quality, physical activity, and sleep pattern) … To investigate the association of physical activity (MET score and steps count), sedentary behaviors (MET score), sleep pattern (sleep duration), diet quality (distribution of total energy, carbohydrate, protein and fat), and light exposure (timing responded to a different type of light) with the glycemic result, the multivariable generalized linear model will be performed… A logistic regression test will be used to examine the association between early and late-night mealtime and diabetes incidence. |
|  | 20b | Methods for any additional analyses (eg, subgroup and adjusted analyses) | Not applicable | Not applicable |
|  | 20c | Definition of analysis population relating to protocol non-adherence (eg, as randomised analysis), and any statistical methods to handle missing data (eg, multiple imputation) | Not applicable | Not applicable |
| **Methods: Monitoring** | | |  |  |
| Data monitoring | 21a | Composition of data monitoring committee (DMC); summary of its role and reporting structure; statement of whether it is independent from the sponsor and competing interests; and reference to where further details about its charter can be found, if not in the protocol. Alternatively, an explanation of why a DMC is not needed | Not applicable | Not applicable |
|  | 21b | Description of any interim analyzes and stopping guidelines, including who will have access to these interim results and make the final decision to terminate the trial | Not applicable | Not applicable |
| Harms | 22 | Plans for collecting, assessing, reporting, and managing solicited and spontaneously reported adverse events and other unintended effects of trial interventions or trial conduct | Section description of risks, pages 12-13, lines 470 to 489. | The CGM system (Freestyle Libre System Pro IQ) is a … Participation in this study is of minimal risk. The medical officers and family medicine specialist will monitor the adverse events of wearing CGM sensors and will provide the clinical support if necessary. However, participants are free to stop at any time during data collection if they feel uncomfortable. |
| Auditing | 23 | Frequency and procedures for auditing trial conduct, if any, and whether the process will be independent from investigators and the sponsor | Not applicable | Not applicable |
| Ethics and dissemination | | |  |  |
| Research ethics approval | 24 | Plans for seeking research ethics committee/institutional review board (REC/IRB) approval | Section ethics approval, Pages 12 to 13, Lines 492 to 498. | A written information sheet about the study protocol will be provided to participants and written consent is obtained during recruitment ... The ethical approval has been obtained from the Medical Research and Ethics Committee (NMRR ID-21-02090-SKX (IIR)) and the Tunku Abdul Rahman University College Ethics Committee (TAR UC/EC/2021/02-3). |
| Protocol amendments | 25 | Plans for communicating important protocol modifications (eg, changes to eligibility criteria, outcomes, analyses) to relevant parties (eg, investigators, REC/IRBs, trial participants, trial registries, journals, regulators) | Section Ethics Approval, Pages 12 to 13, Lines 493 to 495. | Any changes in the study protocol or instruments used shall obtain approval from the ethical committee, followed by a re-consent of the participants whenever necessary. |
| Consent or assent | 26a | Who will obtain informed consent or assent from potential trial participants or authorised surrogates, and how (see Item 32) | Section recruitment, Page 6, Lines 175 to 177.  Section quality control, page 12, Lines 462 to 467. | During the recruitment process, the trained research assistant receives the study description and written informed consent from prediabetic individuals who agree to participate. Dietitians are witnesses to the process.  To ensure that the database accurately reflects the data reported in the questionnaires, a pilot test will be carried out. The research assistant will be trained to carry out the study procedures including obtaining administrative questionnaires, CGM device, 3 days dietary record, and pedometer. A monthly report will be documented after the meeting. A weekly recruitment status will be reported to all co-researchers and supports provided for the recruitment process. |
|  | 26b | Additional consent provisions for collection and use of participant data and biological specimens in ancillary studies, if applicable | Not applicable | Not applicable |
| Confidentiality | 27 | How personal information about potential and enrolled participants will be collected, shared, and maintained in order to protect confidentiality before, during, and after the trial | Section data protection, Page 12, Lines 451 to 459. | All participants will receive a printed information sheet during the baseline recruitment. The data of the consent form will be kept confidential in electronic form and secured with password protection by a trained research assistant. All data evaluated in the baseline and follow-up assessment will be pseudonymously documented. The trained research assistant will enter the data of questionnaires and consent form and a trained registered dietitian will complete the data entry of 3 days dietary records in electronic forms. The consent forms, CGM sensors and questionnaires in both hard copies and electronic forms will be stored in the Tunku Abdul Rahman University College cabinet at least three years (Coulehan and Wells, 2012). They will be disposed of for safety after 3 years. |
| Declaration of interests | 28 | Financial and other competing interests for principal investigators for the overall trial and each study site | Section Declarations, Page 18, Line 649. | The authors declared that they have no competing interests. |
| Access to data | 29 | Statement of who will have access to the final trial dataset, and disclosure of contractual agreements that limit such access for investigators | Section Data analysis, page 13, Lines 502 to 507. | Data profiling and cleansing will be performed by a trained research assistant to assess the data quality and correct various structural errors in datasets, such as misspellings and other typographical errors, wrong numerical entries, syntax errors and missing values. The trained registered dietitian will verify the overall datasets to ensure the overall cleanliness and avoid inconsistent data, duplicate data and irrelevant data appearing in the final datasets. |
| Ancillary and post-trial care | 30 | Provisions, if any, for ancillary and post-trial care, and for compensation to those who suffer harm from trial participation | Not applicable | Not applicable |
| Dissemination policy | 31a | Plans for investigators and sponsor to communicate trial results to participants, healthcare professionals, the public, and other relevant groups (eg, via publication, reporting in results databases, or other data sharing arrangements), including any publication restrictions | Section Ethics approval, page 13, Lines 498 to 499. | The findings of the study will be presented at a conference and disseminated in peer-reviewed journals. |
|  | 31b | Authorship eligibility guidelines and any intended use of professional writers | Section Expected Implications for practice, Page 15, Lines 560 to 569. | According to currently available data, medical nutrition therapy for Malaysian diabetic patients should include meal timing as one of the strategies to manage glycemic control. A study should be conducted to determine the optimal meal timing to manage blood glucose levels, as well as to determine the associations between chronotype and chrononutrition with glucose tolerance. … Future-focused research on these issues can answer these questions. |
|  | 31c | Plans, if any, for granting public access to the full protocol, participant-level dataset, and statistical code | Not applicable | Not applicable |
| Appendices | | | | |
| Informed consent materials | 32 | Model consent form and other related documentation given to participants and authorised surrogates | Section recruitment, page 6, Lines 175 to 181. | During the recruitment process, the trained research assistant receives the study description and written informed consent from prediabetic individuals who agree to participate. The study description is a written information sheet that consists of the study procedure, data collection, data processing, data storage, name and contact information for researchers, potential risks, honorarium and possible withdrawal of consent. The contact list of researchers is also provided in the information sheet. The signed consent form is provided with a randomly assigned ID number. |
| Biological specimens | 33 | Plans for collection, laboratory evaluation, and storage of biological specimens for genetic or molecular analysis in the current trial and for future use in ancillary studies, if applicable | Section glycemic measurements, page 8, Lines 262 to 282. | In a community clinic, participants will receive routine laboratory tests, such as the FBS, OGTT, and HbA1c examination after 8 hours of fasting. This will be followed by giving 75g glucose water within 5 minutes. Consequently, the blood sample will be taken after 2 hours to determine 2hPPG. All blood samples will be analyzed in the community clinic on the same day. The FBS and 2hPPG samples will be analyze using the Dimension^®^ clinical chemistry system (Siemens DF40, United States). The HbA1c samples will be analyzed using Hemoglobin A1c program (Bio-Rad^®^ D-10, United States). The results will be collected and recorded by staff nurses. The blood samples will be kept in refrigerator 4°C for 3 days and disposed accordingly. … The routine next appointment for prediabetic individuals in the community clinic is 6 months to measure the OGTT and HbA1c. |

*It is strongly recommended that this checklist be read in conjunction with the SPIRIT 2013 Explanation & Elaboration for important clarification on the items. Amendments to the protocol should be tracked and dated. The SPIRIT checklist is copyrighted by the SPIRIT Group under the Creative Commons “[Attribution-NonCommercial-NoDerivs 3.0 Unported](http://www.creativecommons.org/licenses/by-nc-nd/3.0/)” license.

1. [↑](#footnote-ref-1)
